# The development and validation of a questionnaire to explore medical students’ learning in a blended learning environment

**DOI:** 10.1186/s12909-021-03045-4

**Published:** 2022-01-03

**Authors:** Rouba Ballouk, Victoria Mansour, Bronwen Dalziel, Iman Hegazi

**Affiliations:** School of Medicine, University of Western Sydney, Sydney, Australia

**Keywords:** Undergraduate medical education, Questionnaire development, Blended learning, MSLQ

## Abstract

**Background:**

A blended learning environment is multifaceted and widely used in medical education. However, there is no validated instrument for exploring students’ learning in a blended learning environment in medical programs. This study aimed to develop and validate an instrument for exploring how medical students learn in an undergraduate medical program that employs a blended learning approach.

**Method:**

Using Artino’s seven step approach, we developed a questionnaire to investigate how medical students learn in a blended learning environment. For pilot testing, 120 students completed this 19-item questionnaire. These 19-items were evaluated for construct and convergent validity across an expert medical education panel. Further item testing was analysed with principal component analysis (PCA) with varimax rotation for item reduction and factor estimation. Hence, validity was thoroughly addressed to ensure the questionnaire was representative of the key focus questions. Cronbach’s Alpha was used for item reliability testing, and Spearman’s Rho was used for the correlation between the questionnaire items and the extensively used MSLQ. Hence, validity and reliability were systematically addressed.

**Results:**

Exploratory Factor analysis identified four factors F1 and F3: Resources: Accessibility & Guidance (14-items), F2: Learning behaviours: Social and Contextual (5-items), and F4: Motivational factors: Intrinsic and Extrinsic Motivation (4-items). Internal consistency and reliability tests were satisfactory (Cronbach’s Alpha ranged from 0.764 to 0.770).

**Conclusions:**

The resulting Blended Learning Questionnaire (BLQ) was determined to be a reliable instrument to explore undergraduate medical students’ learning in a blended learning environment.

**Supplementary Information:**

The online version contains supplementary material available at 10.1186/s12909-021-03045-4.

## Introduction

The implementations of blended learning into medical education curricula have seen many pedagogical adaptations. The combination of face-to-face learning with various online learning resources, referred to as blended learning(BL), has substituted didactic face-to-face lectures [1, 2]. The integration of various learning resources to support students in achieving their learning outcomes has transformed the university’s approach to curriculum delivery [3]. This has facilitated the development of a more student-centred method of learning which encourages students to mature into lifelong learners [4]. Although these pedagogical models are based on educational theory and past experiences, the impact of these adaptations on student learning behaviours has not been well investigated.

Learning is a dynamic process that is initiated by the learner. Students plan and set out their strategies, actively make changes to their goals and adjust their techniques to accomplish their aim. This dynamic participation by the learner ensures that they are constantly reflecting and evaluating the level of success of their implemented strategies in a systematically structured process [5]. Self-regulated learning (SRL) is where the student is behaviourally active and proactive in the learning progression, using goal setting, evaluation and reflection which is driven by motivation to reach their goal [6]. Hence, by implementing these strategies, learners can become successful life-long learners. Medical doctors, in particular, need to keep up with medical progression and thus need to be lifelong learners [4, 7]. To the novice, this process may be challenging and may be dependent on the social supports available and the physical learning environment. These may, in turn, affect the learners’ level of success as each different environment will stimulate the learner in different aspects of their learning. Thus, a successful learner may not exhibit the same level of success in a differently structured mode of delivery due to its nature [8].

The university’s approach to delivering learning material by the substitution of face-to-face lectures with online delivery more recently, due to the COVID pandemic, has encouraged the use of blended learning (BL) more broadly in medical education [9, 10]. The theories of SRL can help us understand the impact of BL strategies on student learning and can help us improve our delivery of the course material. The Motivated Strategies for Learning Questionnaire (MSLQ) is a validated questionnaire that has been used for SRL since 1991 [11, 12]. It is commonly used to help find the relationship between the learner and educator, in relation to their learning technique and strategy in SRL [13]. To our knowledge there is no current questionnaire available that assesses medical students’ SRL in the BL environment. Hence, this paper will focus on the developmental process of a Blended Learning Questionnaire (BLQ) to assess the effect of medical students’ SRL in the BL environment. Consequently, this BLQ would help to obtain valuable data on students’ learning behaviours, ultimately forging a path into curriculum assessment as well as content delivery and by extension, the learners’ environment.

Method.

For the development of the BLQ items we followed the systematic seven step design process for creating high-quality survey scales outlined by Artino et al. [14]. Artino’s systematic, seven-step process includes:

(1) Conducting a preliminary literature review: (2) Conducting focus groups :3. Synthesising the literature review and focus groups: 4. Developing survey items: 5. Conducting expert validation: 6. Conducting cognitive interviews for item interpretation: 7. Conducting pilot testing.

### Data collection Methods

#### Stage 1: Item Development process and content validity

 Firstly, a scoping review was conducted in our previous work which showed it is important to understand medical students’ study habits and how they apply SRL in a BL environment. Consequently, appropriate changes can be made to the design and delivery of the medical curriculum to support SRL and enhance students’ academic abilities. No papers were found that reported on an existing questionnaire that explored SRL in a BL context. From the literature review of SRL and BL, an interview protocol was formulated for the FGD’s to gather information for questionnaire item development (Table 1). Fifteen medical students were recruited from years 1-4 of a 5-year undergraduate medical program. Students varied in age, gender, ethnicity, and tertiary experience to reflect the demographic diversity of the students. Two composite cohort focus group discussions (FGDs) were conducted. The first FGD was for the preclinical years (1 and 2), and the second FGD was for the clinical years of 3 and 4. Participation was voluntary, and anonymity was maintained. These students agreed to review the themes arising from the discussions, helped with the development of questions for the questionnaire and agreed to pilot-test the questionnaire. Each FGD lasted approximately 60 min and was recorded then later transcribed by a professional transcription service.

**Table 1:** Medical group focus group discussion Questions

Please describe yourself as a learner, also including the stage you are currently in.					
: What type of learner are you?					
How do you prepare for learning activities such as lectures or tutorials?					
: Please provide an example?					
How do you approach study during the term, Mid-term and close to the end of term?					
: Does it differ leading up closer to the exams?					
How do you prepare for an exam?					
: What are some of the study changes that you incorporate?					
What influences/or drives your learning pattern?					
: Why does it change throughout the year?					
Of all the resources provided to you by the school of medicine what do you find useful?					
: Why, how and when do you use it?					
: From the resources provided to you by the school of medicine what do you not find useful & why?					
What other resources outside the school of medicine do you use?					

Transcripts were thematically analysed using the six-phase guide framework, stated by Braun and Clark [15–17]. Thematic analysis was conducted by RB and VM separately and then discussed to agree on the final themes see S1 for further details. Following thematic analysis, all authors were involved in developing the questionnaire items provided in the supplementary material, which were categorised into three domains: Learning, Motivational factors, and Content delivery.

#### Expert validation

Following drafting of the items, Face Validity of the questionnaire was evaluated by students who participated in the FGDs to ensure that items addressed the areas identified in the FGD and clearly reflected what was previously discussed. The items were also validated using Convergent Validation with the MSLQ [11, 12]. The BLQ questionnaire contained 19 items adopting a 7-point Likert scale (1= not at all true of me to 7=very true of me), similar to the validated and extensively used MSLQ.

 A panel of 14 members of medical education experts at WSU, consisting of specialists in medical education, clinical medicine and evaluation and assessment, reviewed the drafted items for the BLQ. They were asked to evaluate the preliminary items and provide critical comments on face, content and context validity. The areas that were evaluated through an expert validation process were: representativeness, relevance, clarity, and distribution. The refined questionnaire was then modified further with respect to the experienced local panel members’ feedback in questionnaire development. After undergoing face, content and context validity, the questionnaire was distributed as a Qualtrics™ link to all undergraduate medical students enrolled at Western Sydney University. Of these students, 120 responses were collected in total.

### Stage 2: Statistical analysis and item refinement

Descriptive and inferential statistics were performed using IBM SPSS Statistics version 27 for analysis of the questionnaire items. Exploratory Factor analysis was performed (EFA) with principal component analysis (PCA) and varimax rotation to explore the interrelationships amongst the set of variables that are described in the items and to determine the construct validity of the instrument [18, 19]. The BLQ Internal consistency and reliability were determined by conducting the Cronbach’s coefficient [20, 21]. Spearman’s correlation was also performed to determine correlation between the MSLQ and the BLQ items.

## Results

### Focus group discussions

Through thematic analysis, the FGD data was reviewed following the Braun & Clarke’s (2006) 6-step framework [15, 16]. Analysis of each phase commenced in a nonlinear manner, often going back and forth between phases to visualise any further analytic additions. Three major themes were identified; *learning behaviours, motivational factors and delivery of content*.

### Validity

The emerging themes above were used to develop questionnaire items which were then evaluated for; Face validity, convergent validity, context validity, representativeness, relevance, clarity, and distribution. The MSLQ, a widely used and established tool for SRL and was used as an additional tool to support the validation of the BLQ. Please refer to the MSLQ/BLQ correlation results below. Table 1 demonstrates the extensive validity analyses that were performed all conforming that the items were reflective and appropriate domains.

#### Statistical Analysis

Factor analysis provides statistical guidelines to a researcher in determining construct validity of an instrument [18]. Factor analysis of the BLQ yielded 4 components with restrictions: The 4 component factors were grouped within the major BLQ items as follows: “Resources” encompassing the component factors F1 and F3, “Learning behaviours” encompassing component factor F2, and “motivation” encompassing component factor F4, as depicted in Table 2. The Kaiser Meyer Olkin (KMO) measure of sampling adequacy of 0.655 (p < 0.001), which was above the acceptable limit, indicating that factor analysis was appropriate for this data set [18]. Bartlett’s test of sphericity was significant, (x^2^(546), as (p < 0.001).

**Table 2:** Item analysis in the development of the BLQ

Questionnaire Analysis		
Validity	Face or content Validity	Evaluated by the participants in the FGDs and amended to ensure items are reflective of their discussions
	Content and context validity	14 medical educators inspected the items and confirmed assignment with the appropriate domains.
	Convergent validation	A correlation was found between the developed BLQ sections and the MSLQ. Both questionnaires were categorised into motivation beliefs, cognitive and meta-cognitive strategies, and resource management strategies

**Fig. 1:**
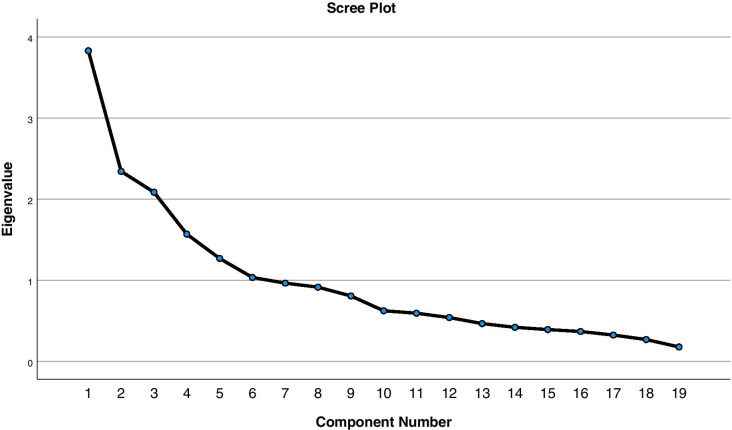
The Screen Plot demonstrating the component Numbers alongside the Eigenvalue identifying the number of factors components

The four factors (F1-F4) with eigen values of 3.83, 2.343, 2.086 and1.569, respectively, accounted for 51.73% of the variance. The factor analysis was appropriate with minimal variance, as it indicated a consistent spread of data with equal distribution, as this is depicted in Fig. 1. Table 3 provides a summary of BLQ results for the 19-items. Together, both the tests, measuring KMO and sphericity, the eigen values all verify factor analysis for the BLQ. Reliability analysis reported an overall Cronbach’s alpha of 0.764 for the 19-item BLQ. Cronbach’s alpha ranged 0.764 to 0.770 Table 4.

**Table 3:** Summary of exploratory factor analysis results for the 19-item Blended Learning Questionnaire (BLQ) Rotated factor loadings Rotated Component Matrixa

	Component			
	F1: Resources: Accessibility & Guidance	F2: Learning: Social and Contextual	F3: Resources: Delivery of content	F4: Motivation: Intrinsic and Extrinsic
BLQ - 13. Accessibility to School of Medicine lectures online enhances my independent learning.	0.796			
BLQ - 16. I use School of Medicine lecture material as a guide for what to learn	0.785			
BLQ - 5. I find the audio-visual online resources provided by the School of Medicine crucial for my learning	0.764			
BLQ - 15. Access to online material off-campus enables me to structure my independent learning	0.663			
BLQ - 3. I am able to consolidate my learning following a small group activity		0.847		
BLQ - 2. I find small group work enhances my understanding about a particular concept		0.774		
BLQ - 9. My study is stimulated by group discussions		0.769		
BLQ - 10. My study habits are influenced by my peers/ social interaction.		0.567		
BLQ - 11. I set up study goals that organise/structure my learning		0.478		
BLQ - 18. Specific external online resources are vital to my independent learning			0.746	
BLQ - 19. I often integrate a variety of medical school and external online resources to support my learning			0.571	
BLQ - 14. I learn more efficiently when I’m able to access online resources using different devices			0.569	
BLQ - 1. I actively seek online resources to prepare my learning materials before a learning activity (tutorial/ lecture/ward			0.564	
BLQ - 6. Flexibility to use a variety of online material motivates my independent learning.			0.549	
BLQ - 4. I find external audio-visual online resources very important to my learning			0.525	
BLQ - 8. My motivation to study increases leading up to exams.				0.723
BLQ - 7. My use of study resources differs leading up to exams.				0.474
BLQ - 12. My study is influenced by the fact that I need to maintain my image (among peers/ supervisors)				0.424
BLQ - 17. Some online resources are efficient because they are well summarised				0.384
Eigenvalues	3.83	2.343	2.086198	1.569138
Percent Of Variance	20.15884	12.33102	10.97999	8.258622
Total variance				51.72847
F1: Guidance and learning support, F2: learning strategy, F3: resources: delivery of content F4: motivational factors “extrinsic and intrinsic “				

**Table 4:** Reliability testing for the 19-item Blended Learning Questionnaire (BLQ)

Factor	No. of items	Item No	Cronbach’s alpha coefficient
**Resources**: F1: Accessibility & Guidance & F4: Delivery of content	8	5, 13, 16, 15. 14, 6, 18, 17.	0.773
**Learning**: F2: Social and Contextual & F5: Structured and Pro-active	5	2, 3,9. 1, 11.	0.721
**Motivation**: F3: Intrinsic and Extrinsic & F6: Social Interactions.	6	8, 4,7,19. 10, 12.	0.554
Overall BLQ	19		0.754

### Spearman’s correlations of the BLQ

Using the Spearman’s correlation test, all 19 items of the BLQ illustrated a range of correlation values (r) greater than 0.3 to greater than 0.5 with adequate significance when matched with similar items in the MSLQ (Table 5). A correlation was found between the developed BLQ domains and the MSLQ, finding that they were both categorised into motivation beliefs, cognitive and meta-cognitive strategies, and resource management strategies.

**Table 5:** Spearman’s rho Correlations between the grouped BLQ and the MSLQ items

BLQ Item Number	MSLQ -item Number	Correlation Coefficient ranging from
BLQ- 1-6	48, 49, 51,53, 70,74,78,81, 75,23, 68, 45, 49, 50, 53, 68, 12, 77, 7, 76, 81	0.314-0.530
BLQ – 7-12.	14, 64, 14, 30, 26, 39, 45, 50, 68, 75,78, 3, 14, 19, 39, 45, 68, 72, 22, 24, 27,43, 49,69, 76,78, 7, 28, 30, 34	0.300-0.519
BLQ – 13-19.	42, 43, 8, 54, 64, 66, 69, 76, 81, 1,4,27, 29, 64, 81, 4, 27, 64, 76, 1, 6, 9, 41, 51, 65, 76, 53.	0.308-0.544
Note: all Correlation coefficients are significant at the 0.05 level (2-tailed).		

## Discussion

Self-regulated learning encompasses the learner’s capacity to actively initiate in their learning and consequently, manage their learning ability [22]. This skill is nurtured by the medical educators resulting in lifelong learners as required by physicians [23, 24]. The modes of delivery within medical education can significantly influence how students learn. Through the incorporation of blended learning modalities, it has impacted previous methods of teaching from traditional to blended mediums, affecting the retention of information in students [1]. It is an important skill for learners to nurture as the student is able to develop a constructive learning environment. This paper has focused on the development and validation of a self-administered questionnaire and is, to our knowledge, the first questionnaire to focus on SRL in a BL environment. Our findings convey that the BLQ questionnaire has satisfactory reliability and validity. The development of the BLQ questionnaire was informed by the literature and the emerging themes of medical students’ focus group discussions. These items reflect the learning habits of the student where motivation becomes the driving force behind the interactive approach to increasing learning outcomes. Items were categorised into three groups, learning, motivation and delivery of content. The themes were extracted from FGDs, and were highlighted by factor analysis as stated in Tables 1 and 2. These factors accounted for 63.86% of the variance.

The theme “Learning”, encompasses students that had described their active learning, detailing how the learner interacts with the learning process. Comparatively cognitive learning refers to the process of active recall and retaining knowledge to learn, where similarly, the learner actively engages with the material [25]. The student’s behaviour changes in-accordance to feedback and the level of success. Hence, behaviourism is a conditioning process of behaviour through positive and negative feedback and implementing them through corrective actions [3, 26, 27]. It was also found that students’ learning is a socially dynamic process varying in context, integrating the role of learning with others. They responded to new situations through their response to feedback, which is a mechanism that is behaviourally dependent. In the learning theory, behaviourism was introduced as a learning theory where it is stimulus dependent [27]. It refers to a feedback mechanism of learning where learners respond to new situations through their experiences to best suit their circumstance.

For the theme, “motivational factors”, students had identified the influential factors for the learner, with the reactive approach to a particular event and in this case, an assessment notification is a primary motivator. Motivation, by setting a short term and a long-term objective, becomes a key modulator, driving time management as another significant factor. Group discussions are another motivating factor that positively place pressure on the student to encourage interaction with their colleagues and share information. This is reflected in the education theory of constructivism which is based on the learners’ experience, where the learners build their understanding and pre-exiting knowledge through reflection of their experience [28].

Learning is a synergistic process, where learners create and test their knowledge relative to others [29]. Hence, social interactions allow students to build a strong foundation in their knowledge. The leaner surroundings and environment were also a significant motivator for the students, with regards to the hospital ward lecture hall, as well as the external factors within that environment. These themes are reflective in the items of the BLQ to evaluate the effectiveness of the medical students learning in a bl environment.

The overall Cronbach’s alpha of 0.75 demonstrates that the BLQ is a reliable instrument [18, 30], and an effective questionnaire to explore SRL in a blending learning environment. Hence, the questionnaire demonstrated adequate internal consistency, as measured by Cronbach’s alpha.

This tool is effective in allowing us to understand the factors that influence medical students’ study habits in response to both internal and external stimuli. This BLQ has demonstrated to be effective as it is congruent with students’ learning behaviours. This was demonstrated by the inclusivity and diversity of the items in allowing students to express the degree to which they relate, to the learning characteristics. For example, we were able to classify pro-active learners by recognising the learning behaviours of consistently reviewing their learning materials prior to classes, actively attending peer discussions to ascertain their level of understanding, as well as scheduling their revision sessions well in advance to these events. This allowed us to recognise the extent that these factors impacted on students’ learning, evaluating the significance of determinants such as their learning setting whether they are in a social peer learning group, on-or-off campus, or whether maintaining a good image with their lecturers and instructors drive their learning, which demonstrated the impact of BL to prompt students to regulate their learning. Hence, we are ultimately able to validate the BLQ as an effective tool that accurately scopes the role of the Blended Learning setting to stimulate changes in the students’ behaviours, as they regulate their learning depending on the availability and type of resources, the learning setting and the medium of content delivery.

## Conclusions

The BLQ emerged to be a valid and a reliable instrument. The validity was determined by a variety of measures and also showed good reliability. This validated questionnaire explores how medical students utilise SRL in the BL environment. This, in turn, can help identify medical students’ learning strategies which can be very useful in curriculum delivery and in developing student support pathways.

## Supplementary Information

**Additional file 1.**

## Data Availability

The datasets collected and analysed during this study are available from the corresponding author on appropriate request.

## Abbreviations

BLQ: Blended Learning Questionnaire
BL: Blended Learning
SRL: Self-regulated learning
