# Supplementary material for: The development and validation of a questionnaire to explore medical students’ learning in a blended learning environment
Source: BMC Med Educ. 2022 Jan 3;22:4. doi: 10.1186/s12909-021-03045-4 (PMC8722320; doi:10.1186/s12909-021-03045-4)
Supplement: Supplementary file 1 — Additional file 1. [file 12909_2021_3045_MOESM1_ESM.docx]

#
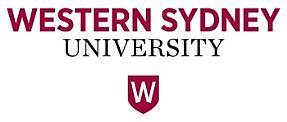
Appendix / Supplementary data

## BLQ Questionnaire

**Bended Learning Questionnaire (BLQ)**

Please rate each item on a scale of 1-7 for relevance by marking the box in the appropriate column

|  | Not at all true of me | Untrue of me | Somewhat untrue of me | Neutral | Somewhat true of me | True of me | Very true of me |
| --- | --- | --- | --- | --- | --- | --- | --- |
|  | **1 2 3 4 5** | | | | | **6** | **7** |
| I actively seek online resources to prepare my learning materials before a learning activity (tutorial/ lecture/ward presentation). |  |  |  |  |  |  |  |
| I find small group work enhances my understanding about a particular concept |  |  |  |  |  |  |  |
| I am able to consolidate my learning following a small group activity |  |  |  |  |  |  |  |
| I find external audio-visual online resources very important to my learning |  |  |  |  |  |  |  |
| I find the audio-visual online resources provided by the School of Medicine crucial for my learning |  |  |  |  |  |  |  |
| Flexibility to use a variety of online material motivates my independent learning. |  |  |  |  |  |  |  |
| My use of study resources differs leading up to exams. |  |  |  |  |  |  |  |
| My motivation to study increases leading up to exams. |  |  |  |  |  |  |  |
| My study is stimulated by group discussions |  |  |  |  |  |  |  |
| My study habits are influenced by my peers/ social interaction. |  |  |  |  |  |  |  |
| I set up study goals that organise/structure my learning |  |  |  |  |  |  |  |
| My study is influenced by the fact that I need to maintain my image (among peers/ supervisors) |  |  |  |  |  |  |  |
| Accessibility to School of Medicine lectures online enhances my independent learning. |  |  |  |  |  |  |  |
| I learn more efficiently when I’m able to access online resources using different devices |  |  |  |  |  |  |  |
| Access to online material off-campus enables me to structure my independent learning |  |  |  |  |  |  |  |
| I use School of Medicine lecture material as a guide for what to learn |  |  |  |  |  |  |  |
| Some online resources are efficient because they are well summarised |  |  |  |  |  |  |  |
| Specific external online resources are vital to my independent learning |  |  |  |  |  |  |  |
| I often integrate a variety of medical school and external online resources to support my learning |  |  |  |  |  |  |  |

**Kindly answer the following questions:**

What are the main resources that support your learning (Medical school and/or external)?

Medical school:

External:

**Thank you**

| **Supplementary Table: Themes, subthemes and exemplar quotes resulting from thematic analysis of FGD** | | | |
| --- | --- | --- | --- |
| **Themes** | **Sub themes** | **Definitions of subthemes** | **Exemplar Student Quotes** |
| Learning | Active learning, Pro-active, Social and Contextual, Behavioural learning, cognitive and visual learning. | **Active learning:** the engagement in the present action, the present interaction of the learner in the learning process. **Pro-active:** (structured): to prepare in advance for events like tutorials, exams, clinical examinations, taking control of the scenario. **Social and Contextual:** that learning is a dynamic process, integrating the role of learning, where learners create and test their knowledge with others by integration the Learning actively from and with others.  **Behavioural:** Refers to a feedback mechanism of learning where learners respond to new situations through their experiences to best suit the circumstance. Your behaviour changes in-accordance to feedback and the level of success.  **Cognitive learning:** The mental action of acquiring knowledge, with the influence of both internal and external factors. It is where Individuals process the information received. **Visual learning:** learners process information better once it is presented to them with graphics. Visual learners prefer reading over listening, and writing over speaking aloud. | “I am a pro-active learner; I take charge of my learning”  “I learn better with hands on experience”  “I found PBL tutorials more useful in comparison to some lectures.” |
| Motivational factors | Assessments, group discussion, reactive approach, goal, interactions with academics and colleagues, time management, feedback, and Environment. | **Reactive approach**: the reaction in response to a particular event that has happened such as an exam or assessment notification. **Goal:** an objective that is set by the learner both short term and long-term goal. **Group discussion**: the interaction of group members where information and their interpretations are shared.  **Interactions with academics and colleagues:** the dynamic flow of communication between members enabling a form of articulation and feedback. **Time management:** The control of time blocking specific activities being mindful to set due dates. **Feedback:** the reaction to a given task, enabling the learner to assess their weakness, target their weaknesses, and continue on feedback.  **Environment:** the learners’ surroundings such as the space they are in, lecture hall, hospital ward, and the external factors within that environment i.e., people in lecture hall. | “I guess I learn pretty well in group settings. Because I feel like having people" "being around group settings, because it helps me to remember things better” “go back and discuss with the tutor or whoever has asked me to do so, I need "someone to bounce off my understanding of the topic” |
| Delivery of content | Accessibility, Online Resources, Condensed resources, Multimodal learning and flexibility. | **Accessibility:** the ability to obtain information before it occurs such as the recorded lectures, or online access of books off campus.  **Online Resources:** the access of learning material from the internet such as study guides. Online access of books off campus **Condensed resources:** A summarised, succinct source of data, collating the information making it more understandable, yet time efficient. This source draws the link between concepts in a time efficient manner.  **Multimodal learning:** the access of the same material using different modes such as interactive websites, e-books, podcasts, YouTube, and Anki cards.  **Flexibility:** the freedom of more access of learning materials on and off campus, any time anywhere accommodates for different type of learners. Learning resources are now more adaptive to the learner. | “I like the c app because u can access from your phone”  “I like the C app access abled access on line, where you get textbook”  “I also use ambos I do a free trial each week as my subscription ran out but I’m doing at free trial every week and definitely the guidelines and flowcharts” |
